# Assessing and Tracking Success of Advancing Age Inclusivity

**DOI:** 10.1093/geroni/igab046.865

**Published:** 2021-12-17

**Authors:** Nina Silverstein, Marilyn Gugliucci

**Affiliations:** 1 University of Massachusetts Boston, Boston, Massachusetts, United States; 2 University of New England College of Osteopathic Medicine, Kennebunk, Maine, United States

## Abstract

Assessment is an important component of advancing age inclusivity on your campus, and the AFU Principles are a useful guiding framework. Assessment helps move the campus from making a commitment to endorse the principles to actually taking stock of current campus practices and movement toward achieving the vision of an age-friendly institution of higher education. To establish a baseline of campus practices, assessment can be done before or after an institution joins the AFU Global Network. Evaluation also follows periodically to assess how well a campus is adhering to the AFU Principles once measurable goals are established and priorities are integrated within an institution’s strategic plan. The toolkit contains examples from multiple campuses of mapping the principles, conducting an audit, doing a photovoice evaluation, holding listening tours, and using the newly developed AFU Inventory and Campus Climate Survey (ICCS).

